# Acting like a doctor: oral case presentation curriculum for medical students

**DOI:** 10.15694/mep.2018.0000028.1

**Published:** 2018-01-31

**Authors:** David Murphy, Nina Gill, Alan Coombs, Rebecca Rooney, Junaid Fukuta, Timothy Reynolds, Justin Morgan

**Affiliations:** 1 North Bristol Academy

**Keywords:** Oral case presentation, communication skills, medical students

## Abstract

This article was migrated. The article was marked as recommended.

Background

Verbal communication is an important element to clinical practice and an integral part of undergraduate medical education. The oral case presentation (OCP) is often used in professional verbal communication and remains commonplace in the clinical setting. The OCP additionally has a complex role in undergraduate teaching.

Methods

We designed a OCP curriculum taking into account reasoning, rhetorical and linguistic mechanisms. Delivered through a content and drama workshop involving a trained theatre actor to 45 pre-clinical, undergraduate medical students at our U.K. institution. Students were assessed objectively at weekly intervals by trained faculty. A paired t-test was performed to determine if the curriculum was effective in increasing OCP scores. Students’ confidence was assessed using Likert scales.

Findings

An overall mean score improvement (M=20.3, SD 14.6, N=45) was significantly greater than zero, t (44) =9.3, two tail p <0.05, providing evidence that the curriculum was effective. A 95% confidence interval around the mean difference in score was 15.9-24.7. Confidence scores for both non-verbal and verbal elements of the OCP improved.

Conclusion

This curriculum led to an improvement in OCP scores and increased our students ‘confidence with this modality of communication. Consideration should be given to incorporating dedicated teaching of the OCP in undergraduate education.

## Introduction

Verbal communication is both an important element to the clinical practice of doctors and an integral part of undergraduate medical education (GMC 2009). Such is the importance of verbal communication that poor communication, especially during handover, has been implicated as a causal factor in up to 80% of ‘serious and preventable adverse events in hospital settings’ ( Bergman et al 2016; Monks and Maclennan 2016). The oral case presentation (OCP) is one method often used in professional verbal communication and remains commonplace in the ward round, clinic and outpatient settings.

In addition to its implications in professional practice, the OCP has a complex role in undergraduate teaching. Firstly, it provides an avenue for student evaluation and simultaneous feedback ( Wiese et al 2002; Lingard et al 2003). Secondly, there is increasing recognition that the OCP also plays a subtler role in facilitating the acquisition of a new identity as medical students progress through their training.

### A Tool for Evaluation

The role of the OCP as a tool for evaluating and giving feedback to students focuses on students’ ability to collect, assimilate and synthesise the relevant information in order to aid the process of diagnosing and managing a specific patient. The OCP may form the basis of informal learning on the wards or in clinic. At our institution, students are formatively assessed on their ability to present an OCP. To the listener, the students’ presentation is a proxy measure for how well the student understands the information they have gleaned from their clerking. If the student is unable to articulate their thought processes and lead the listener through their presentation toward a coherent differential diagnosis, that is taken as a marker that although a student may be able to perform an assessment of a patient, they are unable to understand what their assessment means. The use of the OCP as an assessment tool therefore depends in part on the ‘audience and presenter [sharing] a method of processing information’ ( Wiese et al 2002, p. 212).

Although the processing of information is vital to the OCP, and indeed to medical practice, all too often students are left with little tangible guidance. Instead of simply instructing students to only include relevant detail in the OCP, we need to help students to understand what is relevant. There are several papers looking at facilitating this understanding both through clinical reasoning, and understanding of rhetorical theory ( Haber and Lingard 2001; Wiese et al 2002).

### Socialisation

Social learning theory suggests that ‘a key to novice socialisation is not to learn *from* talk..but to learn *to* talk as a means of acting expertly in a domain’ ( Lingard et al 2003, p. 618). The OCP provides a space for students to learn from and use ‘talk’, and it is therefore partly through the OCP that medical students gain their new identity as doctors ( Bergman et al 2016; Anspach 1988; Monks and Maclennan 2016). Interestingly, students often report finding the OCP to be very difficult ( Bergman et al 2016). This may be due to the complex and multiple learning opportunities the OCP presents; students can easily recognise the role that OCP’s play in verifying clinical knowledge because feedback on subject matter is instantaneous, but the socialisation that students undergo as a part of the OCP is perhaps more occult, yet arguably longer lasting.

The OCP not only allows the listener to evaluate and therefore aid students’ understanding, it also informs the listener of the professional status and competence of the student ( Lingard et al 2003; Bergman et al 2016). The way in which we as the audience of the OCP judge the competence of our students depends primarily on two particular aspects of the presentation; the structure of the presentation, and the language used within it ( Lingard et al 2003; Anspach 1988; Bergman et al 2016; Kroenke 1985). It is the language in particular that plays the greatest role in declaring the socialisation of medical students.

Not only can language give the listener an idea as to how much progress a student has made in their journey to becoming a doctor, language can in fact shape the student’s journey. One controversial linguistic theory, Whorf-Sapiri hypothesis ( Whorf, 1956), suggested that language is more than a tool of expression. This theory proposed that language itself informs our view of the world. This view fell out of favour in the decades after its first publication, but has recently been reviewed, and has gained some traction with a softer version. Whilst designing a study to evaluate the exact relationship between thought and language has proven difficult, some authors have sought to address this problem, and there is now a renewed interest in this area ( Zlatev and Blomberg, 2015; Casasanto, 2008).

Whilst the precise relationship between thought and language is debated in linguistic literature, proponent of social constructivism accept that the socialisation of an individual to a new identity does indeed take place through the acquisition of a new diction ( Mann 2011). Accessing the world of the medical professional depends then, at least in part, in acquiring a new diction in order to share in this particular perspective. The OCP is one method of expressing this new vocabulary, and allows students to signal their entry into the medical world.

It is not surprising, therefore, that students find the OCP difficult. Not only do they feel pressure to display their understanding and analytical processes, but they are also tacitly expected to speak like a doctor, often without explicitly being told how or why. If they are not yet fully inaugurated into the medical community, students’ professional authenticity is undermined in the eyes of the listener, and this is felt by students, although not expressed in this way to them. It is this jarring nature of the OCP that we set out to remedy.

Despite the importance of the OCP to students’ development, teaching is often lacking in this area. Students are noted to rely on a “rule based storage activity.. [and typically use] the same organizational format as their written records” ( Green et al. 2009). Our institution did not offer any formal training on the OCP prior to the development of this short course, reflective of the usual ad hoc trial and error approach described in the literature ( Haber et al. 2001). We therefore designed a method of teaching students about the OCP taking into account reasoning, rhetorical and linguistic mechanisms.

## Methods

The curriculum was delivered to 45 second year medical students from the North Bristol Academy, University of Bristol. The course was run during the summer term as part of a four-week block designed to help the students’ transition from pre-clinical to clinical medicine. During the block, the students were asked to perform full clerking of one patient per week. These clerking’s formed the basis of the OCP’s that were presented in small groups of eight students facilitated by one member of faculty.

As, at the time of writing, no validated marking schemes were available, a marking scheme was developed by two authors (DM, NG) to assess the quality of the OCP. This focused on two areas of the presentation; content and non-verbal skills. The content was subdivided into ‘history’ and ‘examination’. The history subsection included marks for inclusion of certain ‘headings’ such as family history and social history. It also included a Likert scale based on the quality of the history ranging from novice to expert. This challenged the diagnostic reasoning of the candidate and their ability to prioritise information. The examination subsection also included a Likert scale to highlight the need to include pertinent positives and negatives when presenting examination findings. Non-verbal scores were based on the candidates’ ability to perform independently of their notes and engage the listener.

A maximum score of 38 was available for history, 18 for examination and 25 for non-verbal communication. An overall impression score was included giving a maximum score of 86 for each OCP.

All students were assessed prior to any intervention by presenting an OCP to a faculty member who had received training on using the marking scheme. This gave a baseline level of competence with the OCP. A five point Likert scale assessing the students’ confidence with the OCP was also utilised and completed before and after each workshop.

The curriculum began with a workshop on the relevance, structure and content of the OCP. This involved an interactive lecture outlining a structured approach to the OCP. It incorporated discussion on the relevance of OCP’s and a video example of a poor oral case presentation for the students to watch and critique. They were also given a handbook detailing a possible structure of the OCP together with examples of how to address each part of the OCP for common presentations.

A breakaway session then allowed time for the students to video each other presenting cases and critique themselves with the help of the faculty. The video recordings of the presentations helped to provide instant feedback by their peers and faculty to the students regarding the content and delivery of the presentation, thus incorporating the non-verbal element to the OCP.

A subsequent assessment of the OCP was undertaken one week following this intervention when students presented the case that they had obtained in the clinical environment to their peers and a faculty member in a small group format. This OCP was again graded and students were asked to identify their level of confidence using Likert scales.

The second workshop was delivered by a professional theatre actor and director, with experience delivering communication workshops to drama students, medical students, management and other professional groups. This session focused on rhetoric and the use of language between participants in any form of communicative exchange. Firstly, working in pairs, the students were asked to communicate using only physical gestures to guide their partner around the room. This exercise introduced the concept that each new conversation with a new partner develops its own set of ‘rules’ which cannot be transferred. Verbal language was then added back in to their repertoire with ‘yes’ and ‘no’ being used to demonstrate that complex ideas can be communicated through intonation and intent. The final part of the session focused on storytelling and how important information may be lost or altered during an exchange depending on the way the information is communicated in the first place. A third OCP was then assessed one week later (course completion) together with a final Likert scale rating their confidence with the OCP.

Feedback was collected from students with pre- and post- teaching session questionnaires, and separately as part of their feedback for the 4-week long block that this curriculum was placed into.

## Results

Data was collected for a total of 45 second year medical students who took part over a two-month period.

A baseline presentation is compared to presentations made after a dedicated workshop on oral case presentations, as detailed in the methods section. A further assessment is made after completion of the drama workshop/completion of the curriculum. Mean scores are presented in table 1.

**Table 1.** Mean and standard deviations (SD) scores for the OCP’s presented on three occasions during the curriculum.

Assessment subsection	Baseline (mean± SD)	Post-Content Workshop (mean ±SD)	Completion of curriculum (mean± SD)
**History**	18±5	23±4	24±4
**Examination**	6 ± 3	9±2	10±3
**Non-Verbal**	13 ± 4	15±3	15±3
**Overall Score**	39±9	49±8	51±8

Table 2 below details the mean score differences at each assessment during the curriculum together with 95% confidence intervals.

**Table 2.** Mean score differences (with a 95% confidence interval) and p-values.

Overall Score	Mean difference in score (+/- 95% CI)	P Value (* significant)
Baseline vs Post Content Workshop	9.4 (6-13)	<0.05*
Post Content Workshop vs Post Drama Workshop/Completion of curriculum	10.8 (6.1-15.5)	<0.05*
Baseline vs Post Drama workshop/Completion of curriculum	20.3 (15.9-24.7)	<0.05*

A paired t-test was performed to determine if the curriculum was effective in increasing the oral case presentation score. An overall mean score improvement (M=20.3, SD 14.6, N=45) was significantly greater than zero, t (44) =9.3, two tail p <0.05, providing evidence that the curriculum was effective in increasing overall presentation scores. A 95% confidence interval around the mean difference in score was 15.9-24.7. A further breakdown of the differences in scores for each subsection of the oral case presentation is detailed below in table 3, 4 and 5.

**Table 3.** 

History	Mean difference in score (+/- 95% CI)	P Value (* significant)
Baseline vs Post Content Workshop	5.2 (3.5-6.9)	<0.05*
Post Content Workshop vs Completion of curriculum	0.3 (-1.7-2.4)	0.5
Baseline vs Completion of curriculum	5.9 (4.1-7.7)	<0.05*

**Table 4.** 

Examination	Mean difference in score (+/- 95% CI)	P Value (* significant)
Baseline vs Post Content Workshop	2.4 (1.35-3.45)	<0.05*
Post Content Workshop vs Completion of curriculum	1.2 (0.1-2.4)	0.07
Baseline vs Completion of curriculum	3.5 (2.3-4.7)	<0.05*

**Table 5.** 

Non-Verbal	Mean difference in score (+/- 95% CI)	P Value (* significant)
Baseline vs Post Content Workshop	1.4 (0.05-2.75)	0.04*
Post Content Workshop vs Completion of curriculum	0.5 (-1-2)	0.5
Baseline vs Completion of curriculum	1.9 (0.6-3.3)	0.006*

**Figure 1.**
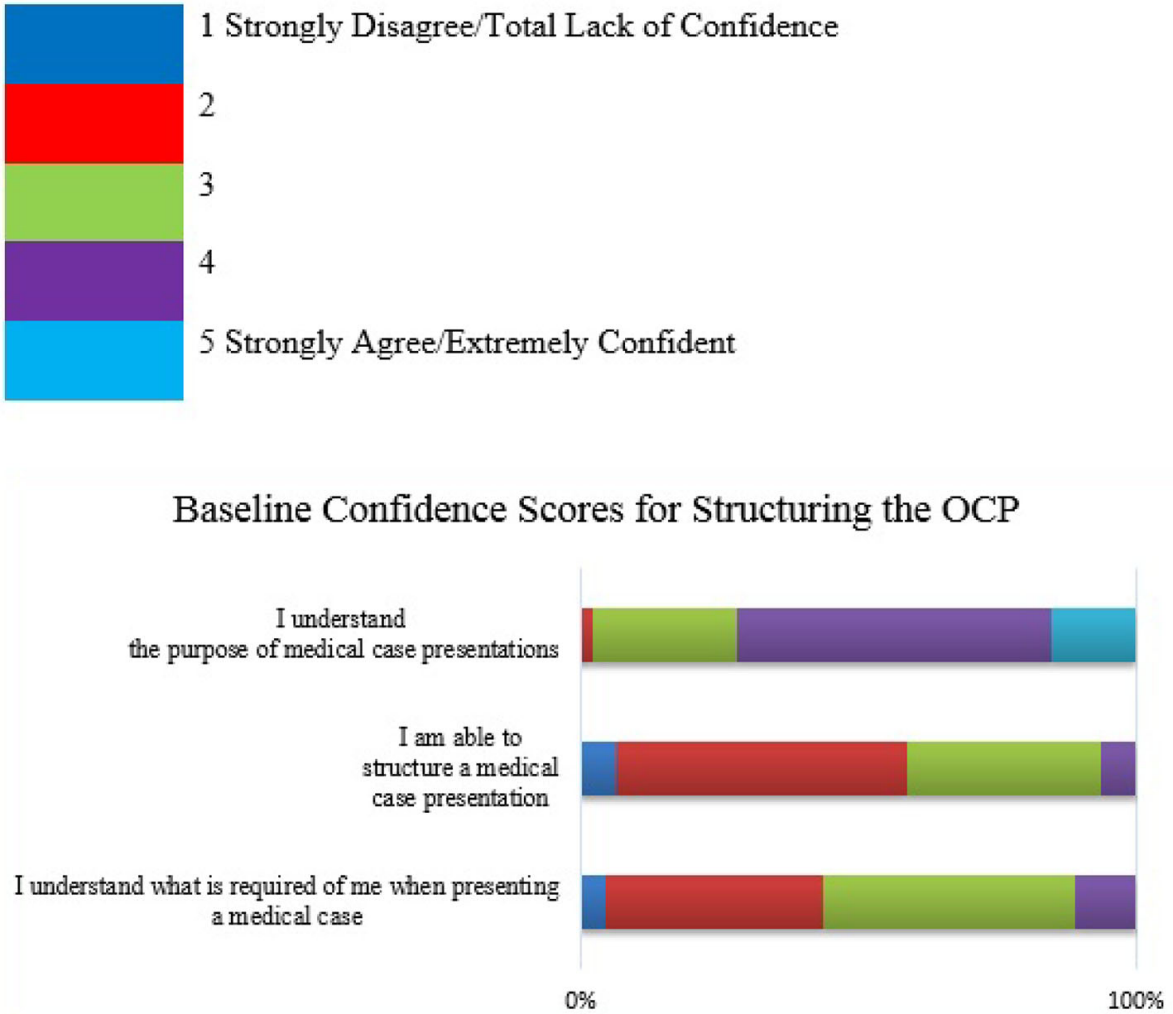
Student Confidence in structuring the OCP (Baseline)

**Figure 2.**
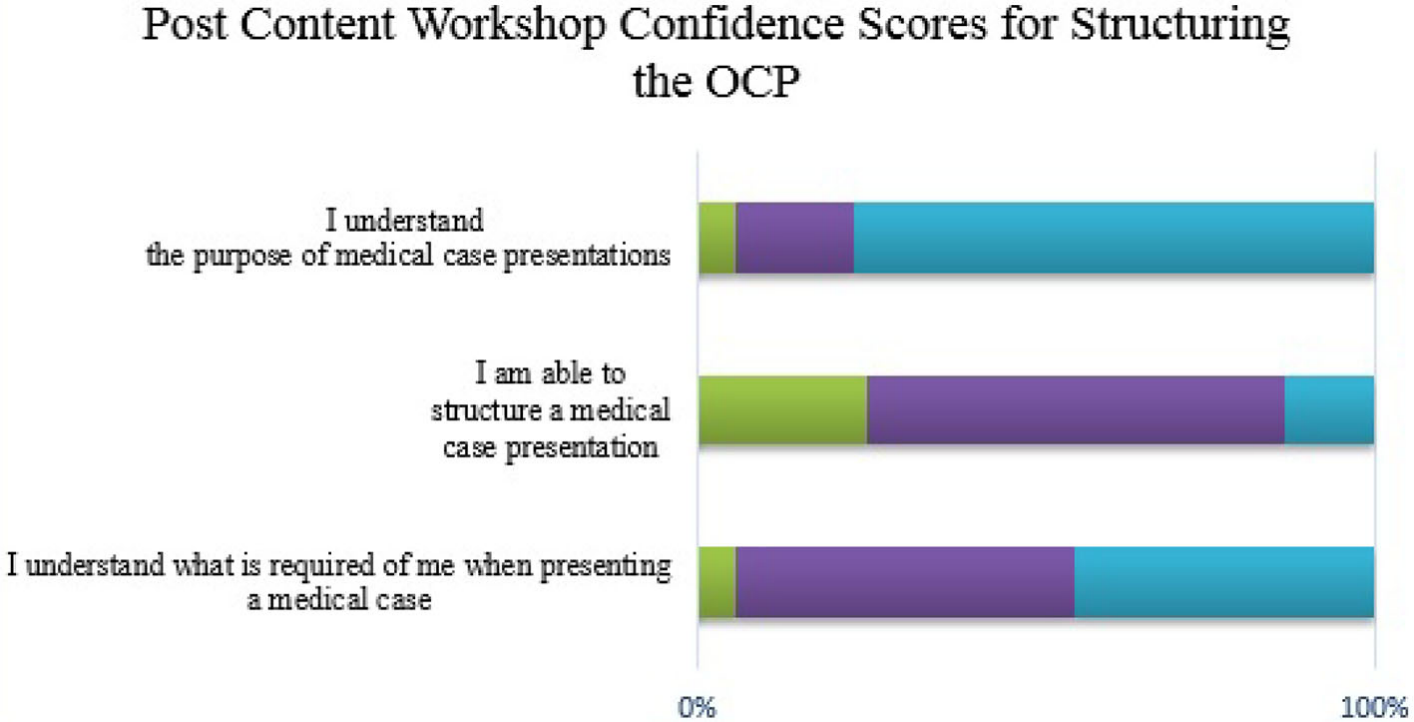
Student confidence in structuring the OCP (post content workshop)

**Figure 3.**
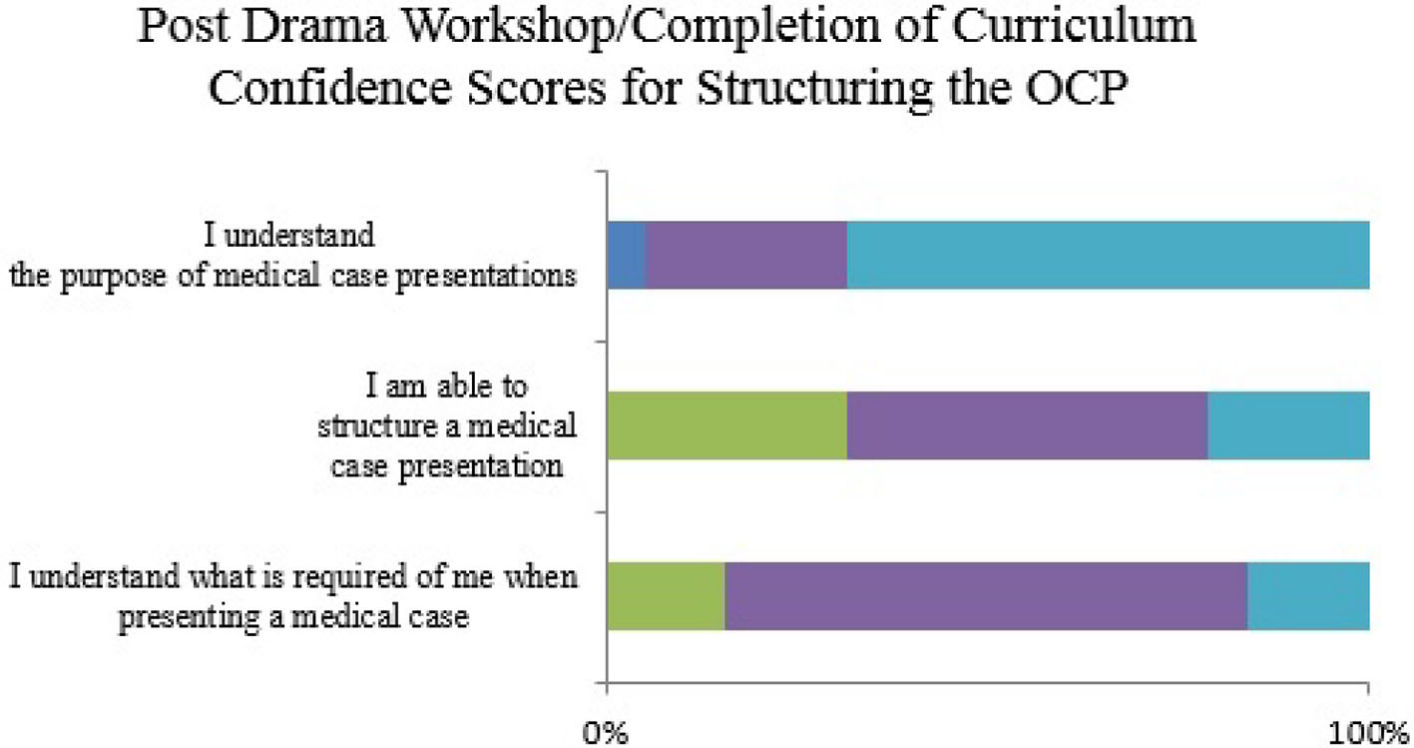
Student confidence in structuring the OCP (completion of curriculum)

**Figure 4.**
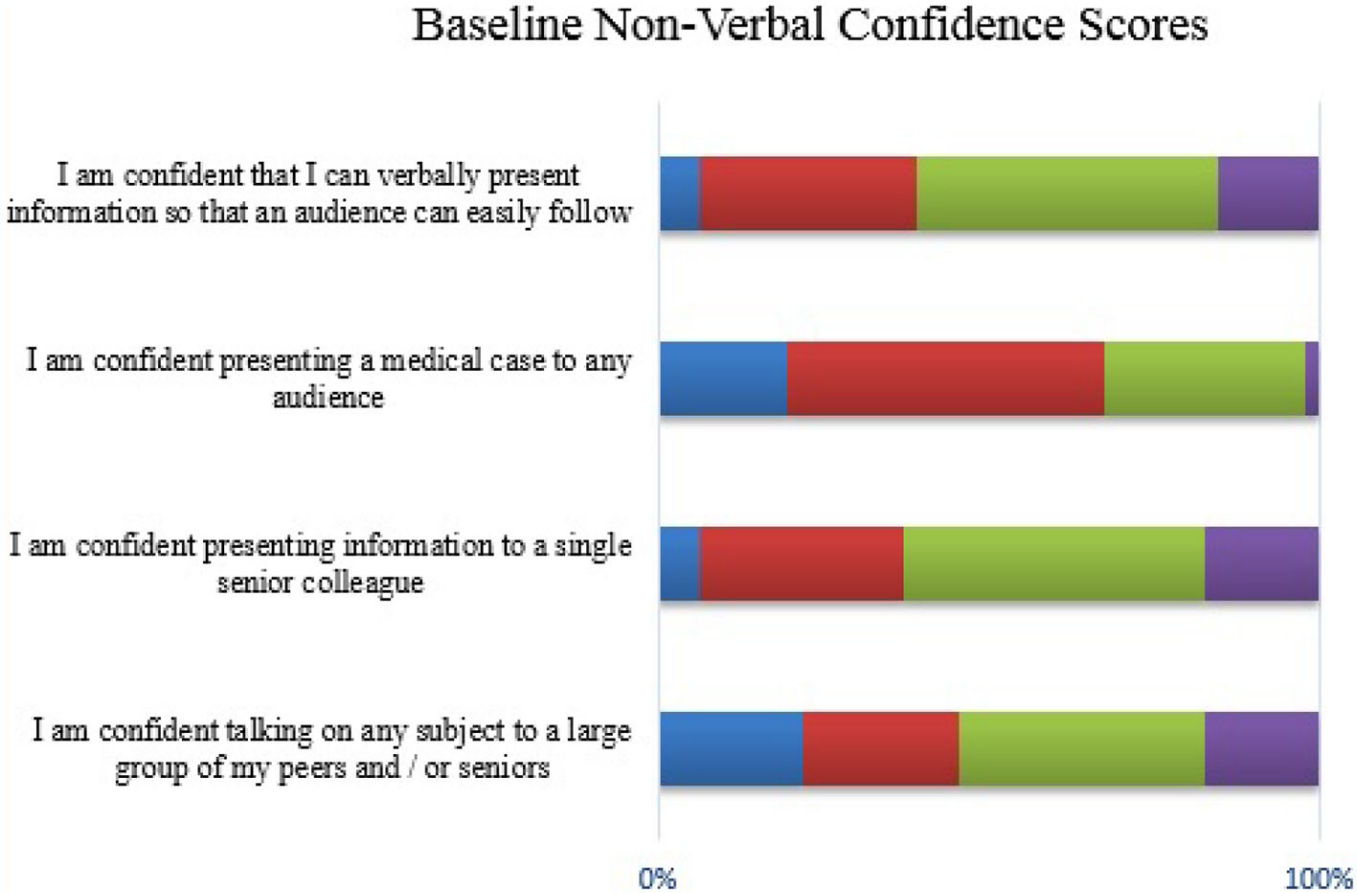
Student confidence with non-verbal OCP communication (baseline)

**Figure 5.**
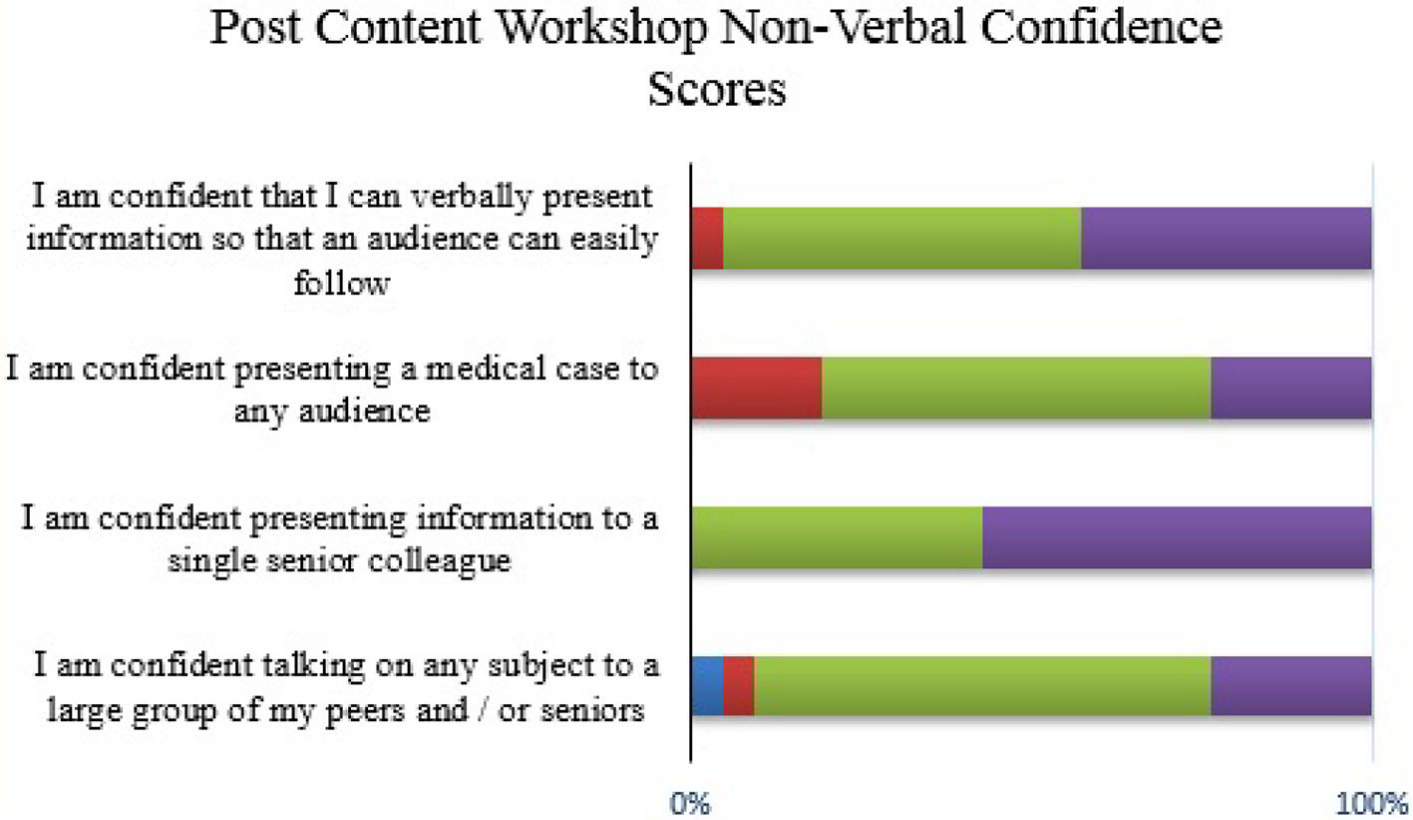
Student confidence with non-verbal OCP communication (post content workshop)

**Figure 6.**
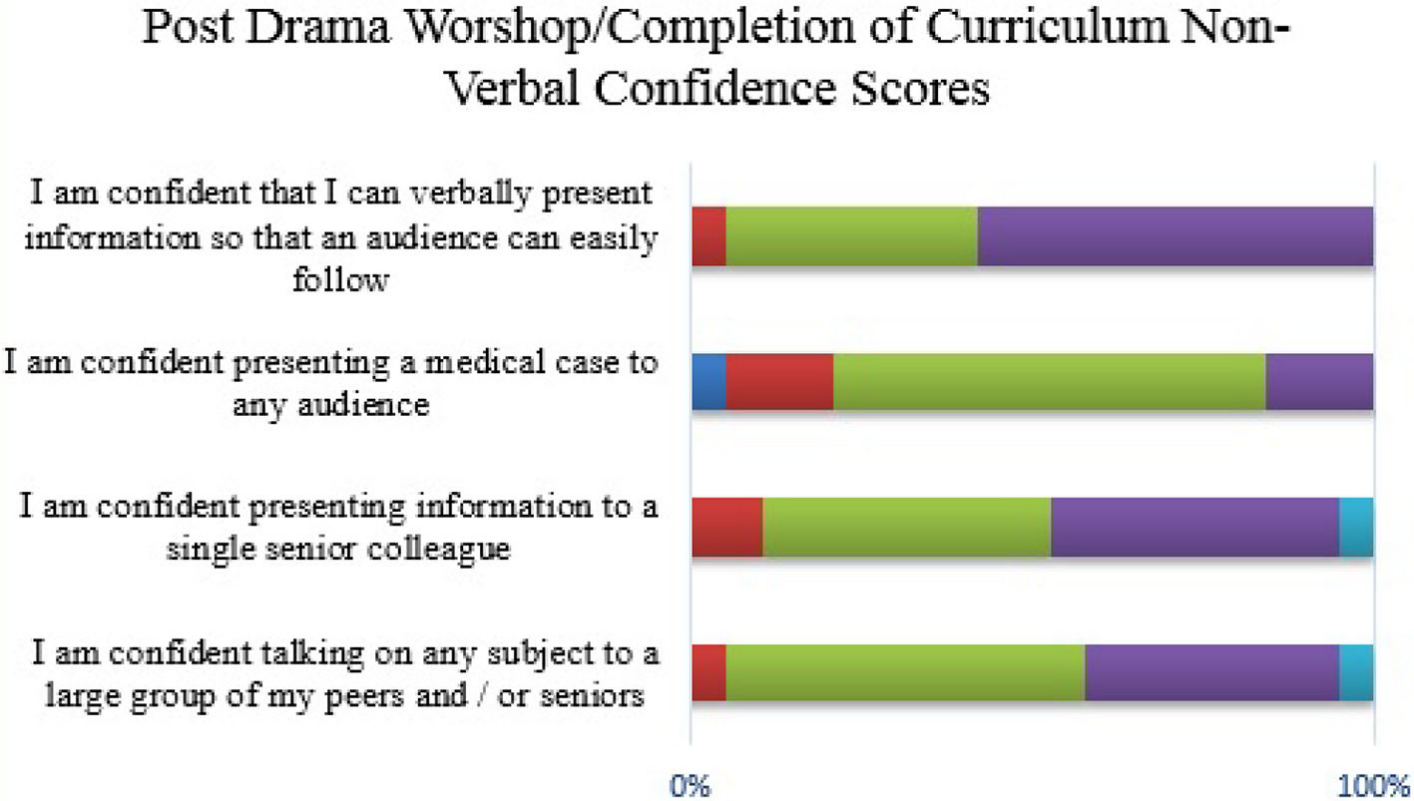
Student confidence with non-verbal OCP communication (completion of curriculum)

Likert scales were used to assess student confidence with regard to the OCP. These are detailed below in figures 1 to 6.

## Discussion

Overall, our results demonstrate statistically significant improvement in objective assessment of the OCP from the start of the course to the end, with a mean difference of 20 points, representing an increase of 23%. This increase came from both the verbal and the non-verbal elements of the OCP.

The improvement in verbal results following the content workshop were expected from the outset, given that this workshop contained a segment addressing recognised approaches to structuring case presentations. The unexpected improvement in non-verbal elements following the content workshop may be explained by the section of the workshop when students were shown a videoed exemplar of a poor case presentation, followed by a discussion and the opportunity to perform the OCP to a partner. We asked the students to film each other on their smartphones, critique each other and watch their own performances back to critique themselves. This was all facilitated by floating faculty members, to aid the students with their assessments of themselves. This may offer an effective and novel way to integrate technology into medical teaching, making use of the easily accessible and high quality video capability.

Interestingly, despite the expectation that the non-verbal scores (eye contact, pace, verbal tics etc.) might increase after the drama workshop, we found that there was no great increase between the content workshop and the drama workshop.

As outlined above, the focus of the drama workshop was more on a global overview of how effective communication takes place between two individuals rather than how to deliver a didactic presentation. Whilst this may go some way to explaining why there was not a great an increase as expected in non-verbal scores over the course, we speculate that the scores would increase further once students were more comfortable with the content element of the OCP.

We noted an overall increase in confidence scores from the beginning to the end of the course, but most of this improvement came from the content workshop. In fact, there was a slight loss of confidence after the drama workshop, which was another unexpected finding. At the same time, the students reported that they had found the drama workshop to be one of the most useful teaching sessions in the entirety of their second year at medical school. We speculate that this may be explained by the idea that the drama workshop tackled an element of communication to which the students had not previously been exposed, and that during this process they found this exploration interesting, and therefore discovered that they had more to learn about successful communication than they had previously realised.

This study had a number of shortcomings. There was no standardised tool available to mark the OCP’s. Facilitators were trained to use the tool developed by the authors, but no formal standardisation of scores was undertaken, so inter-rater reliability cannot be guaranteed.

Another potential pitfall relates the unexpected findings following the drama workshop. The assessment tool that was developed took in to account various objective measures of non-verbal skills, such as maintaining eye contact, but did not capture the aspects of non-verbal communication that are more subtle and difficult to define. Given the overwhelmingly positive and enthusiastic feedback from the medical students on the session and their learning, the assessment tool developed did not capture all the effects that the drama workshop had, and so interpretation of the impact of the workshop based on the parameters set out above may not fully reflect the value of the teaching session.

It is also possible that some of the effect seen could be explained simply by the expected improvement in a skill following practice. This further highlights the need for formalised teaching sessions on the OCP; without a comparison group it is difficult to know how much of an effect simple repetition without intervention would have on students’ scores. Even so, this further emphasises the need to incorporate teaching on the OCP into the curriculum since students’ experience prior to the introduction of this curriculum was ad hoc and very limited, without opportunity for repeated practice.

## Conclusion

Education regarding the OCP is often undertaken in an ad hoc basis despite forming a vital component of medical practice. We have attempted to provide a novel approach involving both the structure and importantly both verbal and non-verbal elements in an effort to produce a curriculum suitable for undergraduate medical students. This was well received by the students with objective improvement and has been adopted by the University for inclusion in the established undergraduate teaching programme.

## Notes On Contributors

All contributors are Clinical Teaching Fellows for the University of Bristol Medical School. Responsible for undergraduate medical education at North Bristol Academy.
