# Supplementary material for: Acting like a doctor: oral case presentation curriculum for medical students
Source: MedEdPublish (2016). 2018 Jan 31;7:28. [Version 1] doi: 10.15694/mep.2018.0000028.1 (PMC10711957; doi:10.15694/mep.2018.0000028.1)
Supplement: Peer review history

# Reviewer report: Reviewer response for version 1

**DOI:** 10.21956/mep.19502.r28830

**Published:** 2018-04-21

**Authors:** Benjamin Hoatson

**Affiliations:** 1 University of Otago

This review has been migrated. The reviewer awarded 2 stars out of 5 A very thought-provoking manuscript. The Oral Case Presentation (OCP) is one of the cornerstones of clinical medicine communication. It is a complex task that requires assimilation and synthesis of information, clinical reasoning, and clear articulation/presentation. Because of this, the OCP poses as a significant barrier for pre-clinical medical students as they transition into clinical medicine. Despite its importance, research into education around the OCP is severely lacking, partly due to the heterogeneity in its format between different medical specialties and its inherently complex nature.Strengths of the study included:• Drama workshop. It was refreshing to see a holistic approach to the OCP that tried to address the content (content workshop) and the delivery of the OCP (drama workshop).• Non-verbal communication assessment. The inclusion of non-verbal communication in the marking scheme of an OCP is consistent with the course design (content and drama workshop) as well as EBM in the assessment of other communication tasks, e.g. history and examinations.• Use of mobile phone to record OCP for peer-peer feedback. The ability to retrospectively watch oneself is an invaluable tool for self-evaluation for the students as well as provides an opportunity for the students to monitor their progress throughout the course.There are several concerns of the study:• Query of results: A statistically significant mean difference between Post Content Workshop vs Post Drama Workshop/Completion of curriculum was stated to be 10.8 in Table 2. This cannot occur if the difference in mean scores of Post-Content Workshop (49) and completion of Curriculum (51) is 2 in Table 1. This discrepancy was also noted for baseline vs Post Drama workshop/Completion of curriculum which was 20.3 in Table 2, rather than 12 in Table 1. This inflated over-estimation of benefits of the study should be amended. • Omission of feedback analysis: Collection of feedback was not explicitly stated in the method section however, the authors unsubstantiated stated “students reported that they had found the drama workshop to be one of the most useful teaching sessions in the entirety of their second year at medical school” and regarding the drama workshop there was “overwhelmingly positive and enthusiastic feedback from the medical students”.• Omission of statistical analysis of Likert Scales of student confidence: Analysis of these results were not reported in the results despite making reference to the interpretation of the data in the discussion “slight loss of confidence after the drama workshop”. This omission is likely because of the negative picture it casts over the utility of the drama workshop.Suggestions:• The inclusion of the omitted results as stated above• The inclusion of a qualitative assessment. I would also echo Subha Ramani within the comments that, assessment of student perception of the course would be invaluable and offer clear insight if there are any benefits of the drama workshop.• Speech/public-speaking coach may be more appropriate than a theatre actor and director. • Publication of the developed OCP marking schemeOverall, the article presents a novel holistic approach (content and nonverbal communication) in medical education around the OCP. This article would be of interest of medical educators involved in preparation courses around the transition of pre-clinical to clinical medicine and educators in clinical skills tutorials, but caution needs to be taken as the data appear to have been incorrectly reported/documented.*This is a student review*

Reviewer Expertise:

NA

No decision status is available

# Reviewer report: Reviewer response for version 1

**DOI:** 10.21956/mep.19502.r28829

**Published:** 2018-02-02

**Authors:** Subha Ramani

**Affiliations:** 1 Harvard Medical School, Brigham and Women's Hospital

This review has been migrated. The reviewer awarded 5 stars out of 5 As an educator who regularly teaches clinical skills, I absolutely enjoyed reading this paper. It is very well written, well referenced. The study design and the curriculum design are excellent. I particularly like the fact that the authors examined both content and non-verbal skills and engaged trainers with drama skills. The authors also describe the study limitations well.I only wish the authors had looked at qualitative outcomes as well and explored what aspects of this curriculum were perceived to be useful to the students and what their perceptions were in general about this educational strategy.Overall, this study and type of curriculum will be useful to all clinical educators

Reviewer Expertise:

NA

No decision status is available

# Reviewer report: Reviewer response for version 1

**DOI:** 10.21956/mep.19502.r28831

**Published:** 2018-01-31

**Authors:** Trevor Gibbs

**Affiliations:** 1 AMEE

This review has been migrated. The reviewer awarded 5 stars out of 5 " What goes around, comes around" Having qualified in Liverpool in 1973, this paper struck specific chords with me; remembering the Friday afternoon sessions when all five years of medical school- in those days 500 students- were invited to the weekly oral case presentations - those students in final year were all expected to present one clinical case- in whatever subject- to the rest of us and then receive feedback from the senior faculty members - the Professors!. It was here I learned my medicine, it was here I thought about role models, the ability to tell a story, the ability to "think on ones feet" and realise that even in final year there was still much to learn. We didn't use statistics in those days to prove the value of such an activity- we all used personal and group reflection- and the realisation that you missed a great learning opportunity if you didn't attend.So, such a long personal reflection to say that I felt that this well written paper is one that curriculum planners should read and as the authors state, consider how we bring back the clinical oral presentations to enhance the many competencies required of our future doctors

Reviewer Expertise:

NA

No decision status is available
